# Work Values of Police Officers and Their Relationship With Job Burnout and Work Engagement

**DOI:** 10.3389/fpsyg.2019.00442

**Published:** 2019-03-14

**Authors:** Beata A. Basinska, Anna M. Dåderman

**Affiliations:** ^1^Faculty of Management and Economics, Gdańsk University of Technology, Gdańsk, Poland; ^2^Department of Social and Behavioural Studies, Division of Psychology, Education and Sociology, University West, Trollhättan, Sweden

**Keywords:** work values, occupational well-being, job burnout, work engagement, Super’s Work Values Inventory, Conservation of Resources theory, police officers

## Abstract

Values represent people’s highest priorities and are cognitive representations of basic motivations. Work values determine what is important for employees in their work and what they want to achieve in their work. Past research shows that levels of both aspects of job-related well-being, job burnout and work engagement, are related to work values. The policing profession is associated with high engagement and a risk of burnout. There is a gap in the literature regarding the hierarchy of work values in police officers, how work values are associated with job burnout and work engagement in this group, and whether work values in police officers are sensitive to different levels of job burnout and work engagement. Therefore, the aim of our study was to examine the relationships between work values and job burnout and work engagement, in a group of experienced police officers. We investigated: (a) the hierarchy of work values based on Super’s theory of career development, (b) relationships between work values and burnout and work engagement, and (c) differences between the work values in four groups (burned-out, strained, engaged, and relaxed). A group of 234 Polish police officers completed the Work Values Inventory (WVI) modeled upon Super’s theory, the Oldenburg Burnout Inventory and the Utrecht Work Engagement Scale. The results show that police officers gave the highest priority to extrinsic work values. Job burnout was negatively correlated with the cognitive intrinsic work values (Creativity, Challenge, and Variety), while work engagement was positively correlated with the largest group of intrinsic work values (Creativity, Challenge, Variety, Altruism, and Achievement), as well as with the extrinsic work values (Prestige and Co-workers). The police officers showed significant differences, between levels of job burnout and work engagement, for intrinsic work values such as Variety, Challenge, and Creativity (large effects), and for Altruism and Prestige (moderate effects). The findings are discussed within the context of the Conservation of Resources theory, which explains how people invest and protect their personal resources, and how this is connected with preferred work values. We conclude that intrinsic work values are sensitive to different levels of burnout and engagement.

## Introduction

Values are viewed as deeply rooted motivations that guide and explain attitudes, standards, and behaviors (Schwartz, 1999). Values can influence how individuals evaluate various events and their importance, and also how they are motivated to undertake activities in different circumstances. Work is an important domain in a human being’s activities and its main function is to provide economic security. However, work also fulfills other psychological functions which lead to growth and learning, and it is also a manifestation of social activity. Work values determine what is important for employees and what they want to achieve in their work (Warr, 2008). So far, research has placed more emphasis on general values, not those of a specific work context. Additionally, more attention was paid to coherence between the general values of the employees and the values preferred by the organization. Thus, in this study we focused on the work value theory of career development by Super (1957, 1980) because the work context is more significant for the proper psychological functioning of employees, and for clarifying their goals and determining their matching for a given type of further education and career development. This life-span theory of career development emphasizes the concept of “role” (e.g., child, student, worker, or being a police officer), and that work values are important in the development of people’s individual role concepts. Super (1975) believed that “educators and personnel workers must look if they want to attend to motivation in ways relevant to the choices and performances of their students and employees” (p. 190). Work values modeled upon Super’s theory reflect different goals that motivate people to work, and are reflected in both extrinsic values *to* work (outcomes of work), and intrinsic values *in* work (those which people seek in their work activity). The context of work and its conditions are generally important for most people in any culture, and it is worthwhile to investigate which work values are given highest priority in order to understand what drives people in their work. For this reason, knowledge about which work values are given priority is invaluable for organizations.

Values represent our highest priorities (“I know what is important and valuable”), and define what is important and valuable for a person within a specific culture and context. Maslow (1987) exemplified “the basic values of our culture” (p. 183) by honesty, humanitarianism, and respect for the individual. Work is for some people, especially for those engaged in higher level occupations (such as in the policing profession), a means of self-actualization, that is, “valued for its own sake” (Super, 1975, p. 191) through finding a life-role. In Super’s life-span theory of career development (Super, 1962, 1970) more than a dozen specific work values are identified. Super (1980) defined these work values as “an objective, either a psychological state, a relationship, or material condition, that one seeks to attain” (p. 130).

Work values are related to work performance and job satisfaction (Chen and Kao, 2012; Tomaževič et al., 2018). However, in some professions organizational effectiveness depends on the respect of these values. This is mainly related to the ethics of these professions (Wang et al., 2018). For example, in the policing profession, responsibility for the security and effective protection of citizens coexists with a variety of interpersonal relations with citizens, as victims or persons violating the law, and is associated with occupational stress. Consequently, the work values preferred by police officers can be important for organizational success.

Work values can evolve during career development and they are related to vocational maturity. Moreover, they may depend on job-related well-being. In line with the Job Demands – Resources theory (Bakker and Demerouti, 2014, 2017), two aspects of well-being are distinguished, job burnout and work engagement. These are rather independent from each other, but correlated. In the work of police officers, there is a high risk of job burnout, whilst high engagement is expected. Past research shows that levels of both aspects of job-related well-being, job burnout and work engagement, are related to work values (Sortheix et al., 2013; Schreurs et al., 2014; Tartakovsky, 2016; Saito et al., 2018). Values are relatively stable over time (Konrad et al., 2000; Jin and Rounds, 2012), but the policing profession is associated with high engagement and a risk of burnout (Talavera-Velasco et al., 2018; Violanti et al., 2018), and the question is whether work values are sensitive to different levels of burnout and engagement.

There is a gap in the literature regarding the hierarchy of work values in police officers, how work values are associated with job burnout and work engagement in this group, and whether work values in police officers are sensitive to different levels of job burnout and work engagement. Applying the Conservation of Resources theory (COR, Hobfoll, 1989, 2011; Hobfoll et al., 2018) may help understand how people with different levels of job burnout and work engagement are motivated to invest and protect their personal resources at work, and how this is connected with preferred work values. Thus, the aim of the study was twofold. First, we aimed to examine the hierarchy of work values in police officers. Second, we aimed to investigate the relationships between work values and both aspects of job-related well-being, job burnout and work engagement, and whether work values in police officers are sensitive to different levels of job burnout and work engagement in a group of experienced police officers.

### Extrinsic and Intrinsic Work Values

From the perspective of human resources management, an important distinction in work is made between extrinsic and intrinsic values (Ryan and Deci, 2000). Extrinsic work values focus on work outcomes for which people are given tangible rewards that are associated with the economic function of work, such as salary, prestige or job security. In contrast, intrinsic values focus on work outcomes that are related to psychological rewards such as recognition, opportunity for growth, and thriving (Bakker and Oerlemans, 2012; Spreitzer and Porath, 2014). Thus, extrinsic or intrinsic work values may lead to a variety of motivations and they require different managerial instruments and practices.

*Extrinsic values* are related to instrumental aspects of work and provide external rewards or satisfaction. They include such values as striving for financial success and high income, job security, opportunities for advancement, status, and power. Based on Super’s life-span theory of career development, Hartung et al. (2010) suggested that some work values such as Management, Workplace, Security, Prestige, and Income are extrinsic values. Further, Leuty and Hansen (2011) have revealed that these work values, and also Achievement, are moderately positively correlated with extrinsic rewards. Lifestyle was also identified as an extrinsic value (Super, 1962). It is possible that this value is sometimes assessed as “style in private life” (and thus as an intrinsic value), and sometimes as appreciation of the people around us, attitude to free time, and the balance between work and home.

*Intrinsic values* are reflected in an inherent psychological satisfaction with work. They include such general values as autonomy, interesting and meaningful work tasks, challenge, variety, emotional intimacy, community contribution, altruism, and personal growth. Hartung et al. (2010) classified Creativity, Challenge, Variety, Achievement, Lifestyle, Esthetics, Autonomy (or Independence), and Altruism as intrinsic values. Work values such as Creativity, Challenge, Variety, and Achievement are moderately positively correlated with intrinsic rewards (Lyons et al., 2006).

Some work values are ambiguous and may be classified as extrinsic and intrinsic simultaneously. It should be noted that Autonomy is related to both intrinsic and extrinsic rewards with similar magnitudes. In addition, Achievement is somewhat ambiguous, but is related more strongly to intrinsic rewards than to extrinsic rewards (Lyons et al., 2006). Hartung et al. (2010) pointed out that Co-workers and Supervision overlap both intrinsic and extrinsic values.

To better understand the role of work values as intrinsic or extrinsic, the terms of the five levels of Maslow’s (1987) hierarchy of needs can be used. Extrinsic values, due to their instrumental nature, are closely related to the basic levels of needs such as physiological, safety and security, and belongingness. Thus, Autonomy, Co-workers, and Supervision are viewed as more instrumental in the work context. In contrast, intrinsic values are more associated with higher levels of needs such as esteem and self-actualization. Hence, the work value Achievement is more reflected in growth and development and, in this way, can be classified as an intrinsic value. Hartung et al. (2010) argued that “in theory, intrinsic values reflect outcomes people seek in work because they are satisfying in and themselves, whereas extrinsic values reflect those outcomes desired because they provide some external reward or satisfaction” (p. 38).

Work values are positively associated with job satisfaction (Dawis and Lofquist, 1984; Rounds, 1990; Judge and Bretz, 1992; Zalewska, 1999; Dawis, 2002; Chen and Kao, 2012; Tomaževič et al., 2018) and with work performance (Swenson and Herche, 1994). Brown and Crace (1996) proposed that work values that are given a high priority are more critical for decision-making than work values given a low priority. This fact may have practical consequences in professions that frequently require appropriate decisions to be made quickly. Some situations in the work of police officers may be very stressful and require critical decision-making, and it is thus desirable to know which high-priority work values are shared by other police officers, at least in a specific culture or district. Furthermore, Ruibyte and Adamoniene (2013) showed that police officers valued work values other than effectiveness and productivity. In this profession the correct balance between extrinsic and intrinsic work values is particularly desirable because they are deeply held driving forces and they form the culture of the organization (Wasserman and Moore, 1988). The work value theory of career development by Super strongly emphasizes the work context, and its application facilitates connecting these values with occupational rewards and other instruments for personnel management.

### Job-Related Well-Being: Job Burnout and Work Engagement

According to the Job Demands – Resources theory, two facets of well-being, reflecting negative and positive indicators, are job burnout and work engagement (Bakker and Demerouti, 2014, 2017). Job burnout is a serious adverse consequence of work in a chronically demanding and threatening work environment. Job burnout has been described as a specific, prolonged reaction of employees to occupational stress (Schaufeli et al., 2009). It is increasingly emphasized that burnout syndrome comprises two main factors: exhaustion and disengagement from work (Demerouti et al., 2003, 2010). Exhaustion is described as a sense of loss of the physical, cognitive and emotional energy required to perform, as a long-term consequence of prolonged exposure to specific working conditions. Disengagement is a state in which a person distances himself or herself from the work, and holds negative attitudes toward the work object, work content and the work in general. Job burnout is correlated with low arousal emotions and with depression (Shirom and Ezrachi, 2003; Bakker and Oerlemans, 2012). Some studies indicate that job burnout is negatively related to intrinsic work values (van den Broeck et al., 2011; Saito et al., 2018). Nevertheless, the orientation toward extrinsic work values was observed in exhausted and disengaged employees (Tartakovsky, 2016; Saito et al., 2018).

Work engagement is one of the most important constructs of positive well-being at work, and of the adult’s happiness (Bakker and Oerlemans, 2012). Work engagement is defined as “a positive, fulfilling work-related state of mind that is characterized by vigor, dedication, and absorption” (Schaufeli et al., 2002, p. 74). It is positively correlated with intrinsic motivation (Schaufeli and Salanova, 2007). Further, an engaged employee differs from an unengaged one in personal resources such as autonomy, optimism, self-esteem, and self-efficacy (Bakker et al., 2008). An engaged employee is also willing to carry out both in-role behavior and extra-role behavior at work (Bakker and Schaufeli, 2008; Zhu, 2013). Moreover, engaged employees are more oriented to intrinsic work values and rewards (Sortheix et al., 2013; Schreurs et al., 2014; Saito et al., 2018).

Police officers are exposed to high work-related stress (Anshel, 2000; McCreary et al., 2017), and it is for this reason worthwhile to investigate the relationships between work values and job-related well-being. Two aspects of job-related well-being were included in the present study: job burnout (negative aspect), and work engagement (positive aspect). Only one study has investigated these relationships (Dyląg et al., 2013). That study, however, used another concept to assess work values, and aimed to investigate the discrepancy between individual and organizational values. It focused on validation of the instrument in a new population, while we have used a well-validated instrument. Dyląg et al. investigated work values, job burnout and work engagement in white-collar workers, while we have investigated these variables in police officers.

### Conservation of Resources Theory and Job-Related Well-Being

In previous studies, attention was paid to the association between job burnout and work engagement as the two facets of job-related well-being (Timms et al., 2012; Moeller et al., 2018). Some researchers focus on activation and pleasure (Russell, 2003; Bakker et al., 2012), while others take a broader approach and focus on energy, pleasure, challenges, and skills (Salanova et al., 2014).

We suggest that different levels of job burnout and work engagement indicate different degrees of the investment (maintaining and collecting), protect and withdrawal of personal resources in the work process. The Conservation of Resources (COR) theory (Hobfoll, 1989, 2011; Hobfoll et al., 2018) provides a useful framework for the conceptualization of these relationships. According to the COR theory, people strive to obtain, maintain, protect and promote resources that are valuable to them. The first principle of the COR theory postulates that the loss of resources is felt disproportionately more than the obtaining of resources. People who are threatened by a potential or actual loss of resources are more motivated to obtain, retain, foster and protect valued resources for future needs. The second principle of the COR theory postulates that people invest resources to conserve possessed resources, gain additional resources and offset the potential loss of resources. Those with greater resources are less vulnerable to resource loss, and they are more capable of organizing resource gain. This theory suggests that resource loss and resource gain occur in a process of “cycles” or “spirals.” A person who lacks access to large stores of resources is more likely to experience further resource loss when demands accumulate, and in this way goes through a downward spiral. In this way, people strive to accumulate resources, and cycles of resource loss tend to have a higher speed than cycles of resource gain. When moderate or massive losses are experienced, a defensive attitude is adopted.

**FIGURE 1:**
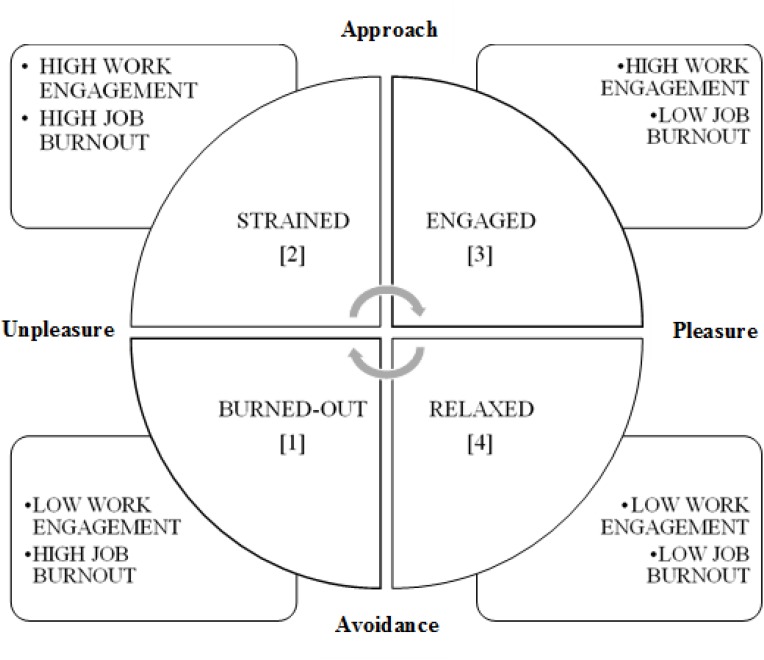
A study model that distinguishes between various combinations of job burnout and work engagement compatible with the Conservation of Resources theory (Hobfoll, 1989, 2011; Hobfoll et al., 2018).

To facilitate understanding of the results from the current study, we used COR theory to create a conceptual model by introducing labels for four groups at different levels of job burnout and work engagement, compatible with the COR theory: burned-out, strained, engaged, and relaxed. Figure 1 presents a theoretical framework of the relationships between different levels of job burnout and work engagement. Two dimensions are defined. The first dimension includes pleasure-unpleasure states, while the second focuses on approach-avoidance with respect to work. These dimensions are related to a person’s affective state and the distribution of his or her personal resources. How can we apply this model on the investigated group? The first group comprises *burned-out* police officers, with a high level of job burnout and a low level of work engagement. This group has depleted personal resources, both energetic and motivational, and can be exhausted and fatigued because the access to resources is limited. The second group comprises *strained* police officers, with a high level of job burnout and a high level of work engagement. These police officers take risks in investing their personal resources because their resources are seriously depleted. In fact, they should protect their resources instead of involving them in the work. This may be a source of tension. The third group comprises *engaged* police officers. These are enthusiastic and characterized by a high level of work engagement and a low level of job burnout. They have strong personal resources and can invest in new ones. The fourth group comprises *relaxed* police officers, with a low level of job burnout and a low level of work engagement. These police officers do not invest their personal resources in work and are satisfied by the current status of their work.

Work values are relatively stable for most adult people, when measured by self-reported inventories (Dawis, 1991; Jin and Rounds, 2012). Work values are deep-rooted and quite stable also in police officers (Hazer and Alvares, 1981), but they may change with time, due to professional experiences, vocational maturity and the consequences of work. Positive experiences and positive consequences of work may result in work engagement, while negative ones may result in job burnout. It is possible that police officers adopt different coping strategies in stressful situations, which may result in their work values changing. The distinctions between the level of job burnout and work engagement interpreted in the COR theory (Figure 1) may shed new light on the priority of work values which are related to the way police officers invest personal resources (dimension of approach versus avoidance) to maintain their affective states (dimension of pleasure versus unpleasure).

### The Current Study

It is necessary to know about the hierarchy of work values held by police officers in order to work actively for their well-being, job satisfaction, career development, intentions to stay in the job, and job tenure. We have pointed out that knowledge about individual work values in police officers is insufficient. Past studies mainly focus on work values among early stage career officers (students and cadets) (Hazer and Alvares, 1981; Zedeck et al., 1981; Sundström and Wolming, 2014; Cuvelier et al., 2015; Kohlström et al., 2017). The associations between work values and other work-related aspects have been relatively well-investigated, but little is known about the relationships between work values and job burnout and work engagement in police officers. Therefore, the aim of the current study was to investigate relationships between work values and job burnout and work engagement in police officers. The following research questions were formulated:

• Which work values are given highest priority by police officers?
• What is the relationship between work values and job burnout and work engagement?
• Are there any differences in work values between police officers with different degrees of job burnout and work engagement?

## Materials and Methods

### Participants and Procedure

Participants consisted of 234 sworn police officers aged between 26 and 58 years (*M* = 35.6 standard deviation [*SD*] = 4.3), and with work tenure between 4 and 30 years (*M* = 12.3 *SD* = 4.5). In this group, 48 were women (21%). The police officers worked as investigators (60%), in the uniformed division (preventive police – 21%), in the logistic division (9%), or with other duties (9%). Most of them were in close relationships (marriage or cohabitation) (89%). They came from a group of police officers undergoing further education for future commissioned police officers at the Police Academy in Szczytno, Poland. Due to this fact, all of them had higher education degrees (93% at Master’s level and 7% at Bachelor’s level). Missing data did not exceed 2%, except for data regarding marital status (2.5%).

### Ethics Statement

According to Polish institutions’ guidelines and national regulations regarding healthy and adult participants, a full ethical review and approval was not required. The study was approved by the Rector of the Police Academy. Before data sampling, the subjects were informed about the aims of the study and the rules for participation (informed consent and the right to information, protection of personal data and confidentiality guarantees, non-discrimination, without remuneration, and the right to withdraw from the study). Participation in the study was voluntary and anonymous. All the participants gave their written informed consent in accordance with the Declaration of Helsinki.

### Instruments

#### Work Values

Work values were assessed by the Polish version (Zalewska, 2000) of the German version (Seifert and Bergmann, 1983) of the Work Values Inventory (WVI), modeled upon Super’s Work Values Inventory (1970). Fourteen of these scales correspond closely to the original English version (Super, 1970). Siefert and Bergman added one value (Promotion) to the original version, and this value was also investigated in the current study. In addition, the work value Leisure was redefined as Lifestyle. The English labels of the work values used here were taken from the recent literature. Using a five-point Likert-type scale from 1 (*Not important*) to 5 (*Very important*), respondents rate the importance of 48 work-related phenomena that constitute 16 scales with three items each. For example, one of the three statements for the Achievement value is: “For me in my professional job, the realization that I have done something very well is…”. Scores range from 3 to 15 for each value, and higher scale scores show that the respondent places greater significance on the corresponding work value.

The User Manual for the WVI (Super, 1970) reports test-retest reliabilities ranging from 0.74 to 0.82 for 2 weeks, and provides sufficient evidence for content, construct, and concurrent validity of the instrument. Seifert and Bergmann (1983), and Zalewska (2000), have shown that both the German version and the Polish version of the WVI have sufficient stability and reliability scores. The internal consistency, measured by Cronbach’s alpha coefficients, was above the lower limit of acceptability (values of 0.60 to 0.70), according to Nunnally (1978), for all work values. Two scales (Autonomy and Lifestyle) had values of Cronbach’s alpha at the lower limit of acceptability, while Management had the largest value (0.86). The WVI scales comprise only three items each, and therefore, Cronbach’s alpha may not be a suitable measure of reliability. Instead, a measure of mean interitem correlation is more appropriate to assess the homogeneity of the scales. A value of mean interitem correlation should exceed 0.30 (Robinson et al., 1991). Values of mean interitem correlations for all work values were satisfactory, with mean interitem correlations between 0.34 and 0.67.

##### Classification of work values by factor analysis

To determine the number of factors to extract from the WVI, we conducted exploratory factor analysis on the single scales, representing a total of 16 variables. In deciding how many factors to retain, we employed parallel analysis (PA) (Horn, 1965; Reise et al., 2000), using SPSS syntax by Hayton et al. (2004). In Table 1 we present the 16 actual eigenvalues from our sample of the 16 scales from the WVI, as well as the average and 95th percentile.

**Table 1:** Actual and random eigenvalues for the 16 Scales from the Super’s Work Values Inventory.

Factor	Actual eigenvalue	Average random eigenvalue	95th percentile random eigenvalue
1	5.774	1.4911	1.5927
2	1.902	1.3771	1.4469
3	1.313	1.2946	1.3693^b^
4	1.024	1.2247^a^	1.2902
5	0.801	1.1701	1.2119
6	0.734	1.1199	1.1619
7	0.628	1.0600	1.1212
8	0.589	1.0079	1.0478
9	0.549	0.9566	1.0131
10	0.504	0.9045	0.9429
11	0.473	0.8569	0.8926
12	0.410	0.8104	0.8492
13	0.367	0.7600	0.8030
14	0.360	0.7073	0.7549
15	0.308	0.6605	0.7070
16	0.265	0.5983	0.6549


*Data for seven multivariate outliers regarding their responses for the WVI scales, were deleted, leaving 227 cases. 81 random dataset were generated on the basis of the same number of scales (16) and cases (227) as in the real dataset used for our factor analyses. ^*a*^This value is higher than the actual eigenvalue, indicating that three factors could be retained according to the rule of “average eigenvalue.” ^*b*^This value is higher than the actual eigenvalue, indicating that two factors could be retained, according to the rule of “95th percentile eigenvalue” (see Hayton et al., 2004)*.

Table 1 shows two different suggestions for retaining the number of factors. In our PA results, both the first two and three actual eigenvalues are greater than those generated by PA (one for the average, and another one, for the 95th percentile criteria). It is therefore difficult to say what is the “correct” number of factors we could choose for the interpretation. However, Harshman and Reddon (1983) and Glorfeld (1995) suggested that PA has a tendency to “overfactor,” and according to Hayton et al. the 95th percentile of each eigenvalue is more conservative than the mean. In addition, the two factor-solution, with one factor comprising externalizing and another one comprising internalizing values is more consistent with Super’s theory and previous research. We therefore decided to retain two factors and interpret them as *externalizing* and *internalizing*.

In the next step, we performed our factor analysis (by principal axis factoring). We applied the practical recommendations of Tabachnick and Fidell (2018). The Kaiser-Meyer-Olkin measure of adequacy (KMO) (Kaiser, 1974) was 0.87 (“meritorious”). Two factors accounted for 41.18% of the total variance (Table 2). An oblique rotation was chosen, because the correlation between the factors was strong (-0.43). After inspection, we labeled Factor 1 as “Extrinsic,” and Factor 2 as “Intrinsic.” Prestige cross-loaded on two factors. Previous findings indicated that Prestige is more correlated with extrinsic than intrinsic reward (Hartung et al., 2010; Leuty and Hansen, 2011). Thus, Prestige was included in the group of extrinsic values. In addition, Achievement cross-loaded on two factors with a somewhat higher loading on Factor 2. In line with Maslow (1987) and Hartung et al. (2010) we included Achievement in the group of intrinsic values (Factor 2).

**Table 2:** Factor loadings, percentage of extracted variance accounted by for each factor, based on principal axis factoring and oblimin rotation with Kaiser normalization of work values in police officers.

Value	Description of the value	Two-factor solution		α	M_iic_
				
		Factor 1 extrinsic	Factor 2 intrinsic		
*Extrinsic*					
Income	Pays well or enables individuals to obtain the things they want	**0**.**81**	**0**.**33**	0.86	0.66
Promotion^a^	Enables individuals to value the possibility of promotion	**0**.**74**	0.04	0.83	0.61
Security	Provides confidence in continued employment	**0**.**58**	-0.22	0.69	0.43
Workplace	Having a work performed in pleasant conditions	**0**.**54**	-0.08	0.83	0.62
Autonomy	Allows or encourages individuals to control the manner in which they perform the work	**0**.**52**	-0.24	0.60	0.34
Lifestyle	Allows people to live the kind of life they choose and to be the type of person they wish to be	**0**.**47**	0.02	0.62	0.36
Prestige	To obtain a high standing in the eyes of others and evoke respect	**0**.**44**	**0**.**44**	0.73	0.47
Co-workers	Having enjoyable interpersonal working relationships with colleagues	**0**.**41**	-0.26	0.84	0.65
Management	Permits individuals to plan and assign the work of others	**0**.**40**	-0.13	0.86	0.67
Supervision	Maintains a collegial relationship with supervisors	**0**.**40**	-**0**.**36**	0.82	0.60
*Intrinsic*					
Challenge	Provides an opportunity for independent thinking and for learning how and why things work	-0.06	-**0**.**78**	0.77	0.53
Creativity	Permits or inspires individuals to invent new things, design new products, or develop new ideas	-0.05	-**0**.**74**	0.79	0.56
Variety	Permits the opportunity to perform different types of job	-0.03	-**0**.**67**	0.74	0.49
Altruism	Enables individuals to contribute to the welfare of others	0.04	-**0**.**58**	0.80	0.58
Achievement	Gives a feeling of accomplishment in doing a job well	**0**.**37**	-**0**.**45**	0.69	0.43
Esthetics	Permits or inspires individuals to contribute to the beauty of the world	0.14	-**0**.**34**	0.83	0.62
Sum of squared loadings		5.21	1.38		
% of explained variance		32.58	8.60		
Factor correlation matrix					
Factor 1		1	-0.43		
Factor 2			1		


**N* = 227; α, Cronbach’s alpha. M_iic_, mean interitem correlation. The values of both α and M_iic_ are calculated on the complete sample of *N* = 234. Description of high scores of work values is adapted from Super (1970). ^*a*^This value was added by Seifert and Bergmann (1983) to Super’s (1970) original 15 values. Values are ordered and grouped by size of factor loadings of the two-factor solution to facilitate interpretation. Factor loadings at least the value of 0.32 (10% of the variance) are in bold*.

#### Job Burnout

Job burnout was evaluated by means of the Polish version (Baka and Basińska, 2016) of the Oldenburg Burnout Inventory (OLBI; Demerouti et al., 2003, 2010). This scale consists of 16 items and measures two aspects of burnout: exhaustion (for example: “After my work, I usually feel worn out and weary”) and disengagement (for example: “Lately, I tend to think less at work and do my job almost mechanically”). Job burnout is the sum of the values for exhaustion and disengagement. The answering format is a four-point scale ranging from 1 (*Strongly agree*) to 4 (*Strongly disagree*). The level of burnout is calculated as the average from the sum of scores divided by the number of items. Higher scores indicate a higher level of burnout. We have used the total OLBI scale score in the work reported here. We chose to do so because we aimed to evaluate the model presented in Figure 1. The descriptive statistics of OLBI in the group examined were *M* = 36.13, *SD* = 6.70, and median [*Me*] = 36, with an internal consistency (Cronbach’s alpha) of 0.79. We did not observe any significant difference in the level of job burnout depending on gender (*t* = 1.20, *df* = 228, *p* = 0.230).

#### Work Engagement

Work engagement was evaluated by means of the Polish version (Szabowska-Walaszczyk et al., 2011) of the short version of the Utrecht Work Engagement Scale (UWES-9; Schaufeli and Bakker, 2003; Schaufeli et al., 2006). This scale measures the overall work engagement in terms of vigor (by the statement, for example: “At my work, I feel that I am bursting with energy”), dedication (“I am enthusiastic about my job”), and absorption (“I feel happy when I am working intensely”). Each item is rated on a seven-point scale ranging from 0 (*Never*) to 6 (*Always/every day*). The average of the sum of scores divided by the number of items gives the level of engagement. Higher scores indicate a higher work engagement. We used the total scale score in the present study, because we aimed to evaluate the model presented in Figure 1. Schaufeli et al. showed that the one-factor model of UWES-9 fits quite well to the data from ten national samples. The descriptive statistics of UWES-9 in the group examined were *M* = 34.42, *SD* = 8.74, *Me* = 35, with an internal consistency (Cronbach’s alpha) of 0.91. We did not find any significant difference between females and males in the level of work engagement (*t* = 0.82, *df* = 228, *p* = 0.412). In this study, job burnout and work engagement were moderately negatively correlated (*r* = -0.58, *p* < 0.001).

### Data Analysis

Descriptive statistics and correlation coefficients between variables were calculated, and the Bonferroni correction was applied (Howell, 1997). Student’s *t*-test for paired samples was applied to calculate the differences between the preferred work values. One-way ANOVA was used to compare differences in the preferred work values between groups. The four groups with different levels of job burnout and work engagement were created (see Figure 1), using the group mean values. The effect sizes were calculated using η^2^. A value of 0.02 for this coefficient indicates a small effect size, 0.06 a moderate effect size, and 0.14 a large effect size (Ferguson and Takane, 1989). The Honest Significant Difference (HSD) Tukey *post hoc* tests for unequal frequencies in the groups were carried out using the STATISTICA 13.1 program. Other statistical analyses were performed in SPSS 25.

## Results

### Descriptive Statistics, Hierarchy of Work Values and Their Associations With Job Burnout and Work Engagement

Table 3 presents details of the hierarchical structure of the work values, grouped into intrinsic and extrinsic work values. The work values measured in the current study fulfilled the criteria for normal distribution.

**Table 3:** Descriptive statistics of work values and their correlations with job burnout and work engagement.

Value	*M*	*SD*	Range	Skewness	Kurtosis	Job burnout	Work engagement
*Extrinsic*							
Supervision	4.51	0.56	2.33–5.00	-1.00	0.57	-0.06	0.12
Workplace	4.36	0.58	1.33–5.00	0.64	0.44	-0.05	0.06
Security	4.26	0.52	2.00–5.00	-0.54	0.76	-0.07	0.13
Co-workers	4.25	0.64	1.33–5.00	-0.59	-0.21	-0.15	0.21*
Prestige	4.11	0.56	2.33–5.00	-0.19	-0.23	-0.12	0.30*
Promotion	3.93	0.65	1.00–5.00	-0.42	1.51	-0.07	0.12
Income	3.91	0.66	1.33–5.00	-0.31	0.35	0.09	-0.09
Lifestyle	3.91	0.57	2.00–5.00	-0.38	-0.05	-0.08	-0.02
Autonomy	3.65	0.60	1.33–5.00	-0.36	0.93	-0.09	0.12
Management	3.08	0.78	1.00–5.00	-0.36	0.009	0.01	0.11
*Intrinsic*							
Achievement	4.20	0.52	2.33–5.00	-0.21	0.01	-0.13	0.23*
Altruism	4.10	0.60	1.33–5.00	-0.35	-0.14	-0.19	0.35*
Variety	3.78	0.63	1.33–5.00	-0.48	0.83	-0.26*	0.38*
Creativity	3.71	0.61	2.00–5.00	-0.18	-0.19	-0.33*	0.47*
Challenge	3.70	0.58	1.33–5.00	-0.22	0.64	-0.28*	0.33*
Esthetics	2.48	0.81	1.00–5.00	0.22	-0.13	-0.12	0.18


*N = 234; ^∗^*p* < 0.05 (after Bonferroni correction 0.05/32 = 0.0016).*

The work values given highest priority by police officers were: Supervision, Workplace, Security, Co-workers, and Achievement. Among the most appreciated work values, only one – Achievement – derived from the group of intrinsic values. The differences between work values in rank 1 Supervision (*t* = 3.79, *df* = 233 *p* < 0.001) and in rank 2 Workplace (*t* = 2.76, *df* = 233 *p* < 0.01) were significantly higher than the next group of work values (Security, Co-workers, and Achievement). Esthetics was not highly valued.

In addition, Table 3 shows correlations between work values and job burnout and work engagement. Table 3 shows that work engagement was more highly correlated with work values than job burnout was. Work engagement was moderately positively correlated with Creativity, Variety, Challenge, Altruism and Prestige.

We noted some difference in evaluation of work values depending on gender. Females valued Achievement (*t* = 3.60, *df* = 228, *p* < 0.001), Supervision (*t* = 3.91, *df* = 228, *p* < 0.001) and Security (*t* = 3.69, *df* = 228, *p* < 0.001) as more important than males did. These effect sizes, measuring by Cohen’s *d* coefficient, were medium (ranged between 0.62 and 0.70). Females also more appreciated than males Altruism (*t* = 2.10, *df* = 228, *p* < 0.05), Prestige (*t* = 2.38, *df* = 228, *p* < 0.05) and Co-workers (*t* = 1.98, *df* = 228, *p* < 0.05). However, these effect sizes were small (ranged between 0.36 and 0.39).

### Examining the Conceptual Model: Differences Between Groups

**FIGURE 2:**
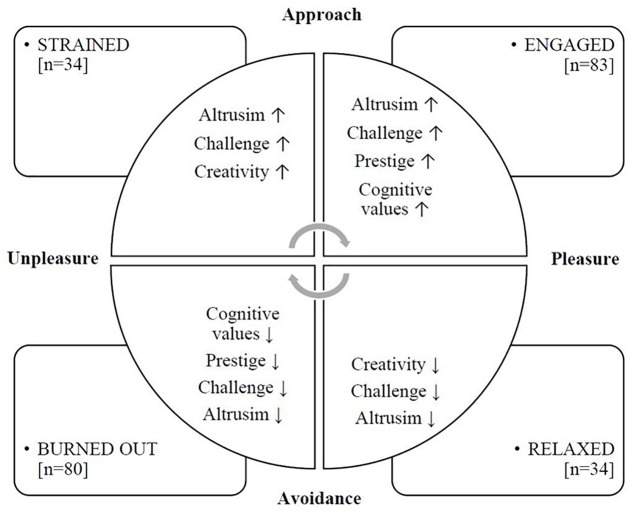
Work values among four groups of Polish police officers (*N* = 231): An application of a model, presented in Figure 1. It distinguished between various combinations of job burnout and work engagement.

Four groups with different levels of job burnout and work engagement were formed by grouping the police officers according to their mean score on OLBI and UWES-9 (Figure 2). Two groups were relatively large: 36% of police officers were placed into the engaged group and 34% into the burned-out group. The strained group and the relaxed group each consisted of 15% of the police officers.

Before applying *post hoc* tests, 6 of the 16 work values differentiated significantly the police officers with respect to job burnout and work engagement. The effect sizes measured by η^2^ coefficients were small for Achievement, moderate for Prestige and Altruism, and large for Variety, Challenge and Creativity. Table 4 presents the statistics relating to group differences.

**Table 4:** ANOVA summary table: work values among four groups of police officers.

Values	Burned out (*n* = 80) [1]		Strained (*n* = 34) [2]		Engaged (*n* = 83) [3]		Relaxed (*n* = 34) [4]		*F*	*p*	η^2^	*Post hoc* (Tukey’s HSD)
								
	*M*	*SD*	*M*	*SD*	*M*	*SD*	*M*	*SD*				
*Extrinsic*												
Prestige	3.97	0.62	4.22	0.51	4.25	0.50	3.99	0.5	4.51	0.004	0.06	[1] < [3]^∗∗^
*Intrinsic*												
Achievement	4.12	0.55	4.26	0.48	4.32	0.52	4.08	0.46	2.86	0.038	0.04	–
Altruism	3.91	0.66	4.21	0.48	4.33	0.46	3.83	0.66	10.35	<0.001	0.12	[1] < [3]^∗∗∗^
												[2] > [4]^∗∗^
												[3] > [4]^∗^
Variety	3.51	0.68	3.82	0.55	4.06	0.56	3.73	0.44	12.00	<0.001	0.14	[1] < [3]^∗∗∗^
Challenge	3.49	0.61	3.69	0.62	3.98	0.48	3.54	0.48	11.64	<0.001	0.13	[1] < [3]^∗∗∗^
												[3] > [4]^∗∗^
Creativity	3.46	0.58	3.8	0.62	4.05	0.47	3.4	0.56	20.59	<0.001	0.21	[1] < [3]^∗∗∗^
												[3] > [4]^∗∗∗^
												[1] < [2]^∗^
												[2] > [4]^∗^


**N* = 231; ^∗^*p* < 0.05, ^∗∗^*p* < 0.01, ^∗∗∗^*p* < 0.001; only results that were significant before applying *post hoc* Tukey’s HSD tests are shown.*

Table 4 shows that burned-out police officers were systematically lower on the presented work values than those who were relaxed. Strained police officers were higher than relaxed police officers on Altruism and Creativity, while the relaxed police officers were lower on these values than those who were engaged. The effects of group differences in Variety and Creativity were large, while the effects on Altruism and Prestige were moderate. After applying the HSD Tukey test, the mean difference between the groups on Achievement was no longer significant and the effect was small.

## Discussion

The results presented here show that: (a) police officers gave the highest priority to the following work values: Supervision, Workplace, Security, Co-workers, Achievement; (b) job burnout was significantly negatively correlated with the intrinsic work values Creativity, Challenge, and Variety, while the magnitude of the correlation was moderate; (c) work engagement was significantly positively correlated with the intrinsic work values Creativity, Variety, Altruism, Challenge, and Achievement, as well as with the extrinsic work values Prestige and Co-workers; and (d) there were significant differences between police officers with different levels of job burnout and work engagement for intrinsic work values such as Variety, Challenge, and Creativity (large effects), and for Altruism and Prestige (moderate effects).

### Hierarchy of Work Values in Experienced Police Officers

The values given highest priority by police officers in this study were Supervision, Workplace, Co-workers, Security, and Achievement. All, with the exception of Achievement, derived from the group of extrinsic values. These values may be regarded as an important key for many successful organizations, and they are related to the five most basic resources in an organization (such as a place to work, the right equipment, money to pay the bills, and the right people working there). Previous studies in healthy and well-educated groups showed that men prefer extrinsic values and that they are more monetary and prospect oriented (Furnham and MacRae, 2018; Guo et al., 2018). Our sample comprised of 80% males, and this may partly explain their preference of extrinsic values, regardless the policing profession. These extrinsic values may also be regarded as lower-order needs, the need of security and the need to belong, as identified in Maslow’s (1987) hierarchy of needs. Previous research has shown that such work values raise intrinsic motivation significantly and lead to people persisting longer with a challenging task willingly, reporting greater interest in the task, becoming more engrossed in it, performing better on it, and spontaneously expressing greater enjoyment of and interest in it (Carr and Walton, 2014). To summarize, the police officers gave a high priority to having an understanding and respectful manager, the possession of good coworkers, friction-free collaboration with others, the certainty of their job, and the security of their current position. Police officers almost always work together, and it was not unexpected that they gave a highest priority to a fundamental basic value – the need to belong.

The priorities given to various work values may differ between different cultures, due to individual differences or the availability of resources in a particular police district or country (this is the case also for Maslow’s hierarchy of needs). Ruibyte and Adamoniene (2013) pointed out that a change in values may occur over time in post-transformation (that is after the fall of the iron curtain and the transformation to market-based capitalism) countries such as Lithuania, because of rapid changes in society, increased competition, and thus, more demanding work conditions. The current study was performed in Poland, which is a post-transformation country. These studies differ in the methods used and this means that it is not easy to compare the results. The police officers studied by Ruibyte and Adamoniene were asked to describe their beliefs about organizational values. They replied that clear time limits, the achievement of good performance, professional development, security, integrity and formality were the beliefs that were most highly valued in the police organization. A study of first-year student police officers in Sweden (Sundström and Wolming, 2014) showed that altruistic values were most important, which contrasts with the results presented here: altruistic values were among the work values given medium levels of priority (Table 3). It is possible that the differences are related to the duration of work tenure. Sundström and Wolming studied student police officers, whereas we have studied experienced police officers. More research on the work values of police officers in different countries is needed.

### Work Values and Their Association With Job Burnout and Work Engagement

A higher level of job burnout and a lower level of work engagement were significantly related to some of the intrinsic work values. This was expected because these two aspects of job-related well-being are correlated with each other and also with an intrinsic motivation (Schaufeli and Salanova, 2007; Maslach and Leiter, 2008; Sortheix et al., 2013; Schreurs et al., 2014; Saito et al., 2018). Furthermore, a higher level of work engagement was correlated with two extrinsic values, Co-workers and Prestige. Employees that are more engaged are more willing to perform an occupational role in a social environment (Bakker and Schaufeli, 2008; van den Broeck et al., 2011).

Three intrinsic work values (Creativity, Challenge, and Variety), closely related to intrinsic rewards, were positively correlated to the level of work engagement, and negatively correlated to the level of job burnout (Table 3). These values are theoretically related to the impairment of cognitive functions (such as frequent switching between tasks, an inability to update activity, inhibition of certain cognitive functions, impairments in sustained and controlled attention, poor long-term and short-term memory, and poor working memory) (Deligkaris et al., 2014; Golonka et al., 2017; Sokka et al., 2017). These relationships suggest that job burnout can be associated with cognitive impairment, which is compatible with findings presented by Deligkaris et al. (2014). Deligkaris et al. showed that people who are burned out have impaired cognitive functions, such as executive functions, attention and memory.

### Differences Between Work Values in Burned-Out, Strained, Engaged, and Relaxed Police Officers

First, we discuss the two largest groups, *burned-out* and *engaged*, according to our conceptual model. Our results (Table 4 and Figure 2) show that the *burned-out* police officers and *engaged* police officers had the largest difference in the mean values of intrinsic values. This is consistent with results of previous studies regarding other groups than police officers (Timms et al., 2012; Salmela-Aro and Read, 2017; Moeller et al., 2018; Schult et al., 2018). These studies suggest that job-related well-being decreases as less priority is given to intrinsic values (Tartakovsky, 2016; Saito et al., 2018). In particular, the group of three work values: Creativity, Challenge, and Variety, was less preferred. As mentioned above, people who are burned out can have cognitive deficits and they need to protect their depleted mental resources more. Further, the *burned-out* police officers gave lower priority to Altruism and Prestige than *engaged* police officers gave. It is not surprising that this group gave less priority to intrinsic work values, since job burnout is characterized by a lack of cognitive and mental energies (Demerouti et al., 2010; van den Broeck et al., 2011). Police officers in this group had neither the power nor the desire to get involved in work-related social relationships or social comparisons. One aspect of job burnout is disengagement, which is expressed in psychological withdrawal from the task, clients and work in general. It is not known whether this withdrawal is an adaptive mechanism to cope with the excessive occupational stress that results in feelings of exhaustion (Hobfoll and Shirom, 2001; Arble et al., 2018). It may also indicate that the police officers limit their investment of resources and adopt a defensive attitude in order to protect themselves.

The *Relaxed* police officers, who were neither burned-out nor engaged, gave a lower priority to Altruism than the *strained* police officers, and they gave a lower priority to Challenge than *engaged* officers. They valued Creativity less than those who were *engaged*, and less than those who were *strained*. These findings are compatible with Hobfoll’s (1989) COR theory. Specifically, the level of engagement determines how much of own resources are invested in the process of work. Relaxed employees invest fewer of their own resources because work in general can be less valuable to them. Thus, they are also called unengaged or unfulfilled in the work and even apathetic (Timms et al., 2012; Moeller et al., 2018; Schult et al., 2018). Our findings are compatible with results obtained by Salanova et al. (2014), and introduce a new aspect: we have included work values in our model (Figure 2). Salanova et al. validated a theory-based classification of employees’ well-being, using a classification of participants similar to that we have used, and identified a group who gave low priority to work. They denoted this group as “working from 9 a.m. to 5 p.m.,” and it corresponds to our *relaxed* group.

The results of the study demonstrated that *trained* and *engaged* police officers showed no difference in their preferred work values. These groups were characterized by similar levels of work engagement (Figure 2). Past research shows that engaged employees are full of empowered (Timms et al., 2012; Moeller et al., 2018). In contrast, strained employees experience job burnout and engagement simultaneously and that they therefore can be considered as striving, frustrated or being under pressure (Timms et al., 2012; Salmela-Aro and Read, 2017; Schult et al., 2018). It can be assumed that a higher level of engagement is conducive to investing personal resources, irrespective of their wealth or poverty (Violanti et al., 2018). In addition, creativity was more important for the police officers who were *strained* than it was for *burned-out*. Results of previous studies have suggested that creative and challenging work is needed in order to satisfy people’s need for growth, learning and development (Ryan and Deci, 2000; Paterson et al., 2014). This result is also compatible with Hobfoll’s (1989) COR theory, which states that some burned-out employees want to invest and thrive, despite the burnout. The engagement of employees is a fundamental factor in building a competitive advantage of an organization on the labor market (Paterson et al., 2014).

### Limitations and Strengths

This study has some limitations. First, it has a cross-sectional design, which does not allow causal directions of the relationships between work values and job burnout/work engagement to be formulated. However, it is theoretically well-proven that preferred work values are closer to personal dispositions (Jin and Rounds, 2012) and have a higher priority. In contrast, well-being is a consequence of work (Bakker and Oerlemans, 2012). Second, the way in which data were collected may have given an unrepresentative sample. The participants were, however, from several Polish districts, which increases the probability of obtaining a representative sample. Third, males outnumbered females, and this imbalance may have affected the results. The number of women in the study (about 20%) was, however, higher than the number of women among the Polish police officers (14%, data from the National Police Headquarters). Fourth, we have not investigated the stability of work values. Work values, however, are relatively stable (Dawis, 1991; Jin and Rounds, 2012; Leuty, 2013). It is easy to assume that work values are dynamic and flexible, as they are influenced by environmental factors (such as education, change of socioeconomic status, change in marital status), and as employees are exposed to work-related trials (such as experiences during working life, consequences of the work carried out, and change in occupation). Furthermore, employees experience rapidly changing societies, increased competition and individualism, and, again, it is easy to assume that work values change with time. However, only few studies have shown that work values are influenced by experiences and by environmental factors. Hazer and Alvares (1981), for example, showed that organizational entry and assimilation resulted in work value stability in police officers.

Police officers are extremely important for a properly functioning society and for citizen security. Taken together, the hierarchy of work values, relationships within the theoretical model, and the discovery of significant differences between the groups provide information that managers should consider when providing career assessment for police officers, and when support them. It is important for managers to identify police officers who show early signs of job burnout, in order to provide work resources and managerial support, which lead in turn to satisfied co-workers and role clarity. Future research should focus on similar variables in the populations of police officers in other countries, in order to determine how general our results are.

## Conclusion

To conclude, we have examined work values of experienced police officers and identified those given a high priority. We have examined also the relationships between work values and two important aspects of job-related well-being, job burnout and work engagement. The results show that extrinsic work values were given high priority. In contrast, intrinsic work values are more sensitive to different levels of job burnout combined with different levels of work engagement. The burned-out group and the engaged group were the most different in terms of intrinsic work values. Three of these intrinsic work values (Creativity, Challenge, and Variety). that are theoretically related to cognitive processes, were positively correlated with work engagement, and negatively correlated with job burnout. Police organizations should be aware that work values are important aspects of working life and thus should be taken into consideration during the selection and assessment process throughout the vocational career.

## Data Availability

All datasets generated for this study are included in the manuscript and/or the supplementary files.

## Author Contributions

BB designed the study, performed the research, analyzed the data, and wrote the manuscript. AD designed the study, analyzed the data, and wrote the manuscript.

## Conflict of Interest Statement

The authors declare that the research was conducted in the absence of any commercial or financial relationships that could be construed as a potential conflict of interest.
